# GigaDB: a redesigned repository for data publishing and management

**DOI:** 10.1093/gigascience/giag047

**Published:** 2026-04-16

**Authors:** Xiaoqiang Li, Cong Hua, Qian Yue, Zhiyong Li, Jiawei Tong, Ziheng Luo, Tao Yang, Lijin You, Hongfang Zhang, Dongni Ma, Xiaofeng Wei, Hongling Zhou

**Affiliations:** BGI Research, Shenzhen 518083, China; Guangdong Provincial Genomics Data Center, BGl Research, Beishan Road, Yantian District, Shenzhen 518083, China; BGI Research, Shenzhen 518083, China; Guangdong Provincial Genomics Data Center, BGl Research, Beishan Road, Yantian District, Shenzhen 518083, China; Guangdong Polytechnic Normal University, Guangzhou 510665, China; BGI Research, Shenzhen 518083, China; Guangdong Provincial Genomics Data Center, BGl Research, Beishan Road, Yantian District, Shenzhen 518083, China; BGI Research, Shenzhen 518083, China; Guangdong Provincial Genomics Data Center, BGl Research, Beishan Road, Yantian District, Shenzhen 518083, China; BGI Research, Shenzhen 518083, China; Guangdong Provincial Genomics Data Center, BGl Research, Beishan Road, Yantian District, Shenzhen 518083, China; BGI Research, Shenzhen 518083, China; Guangdong Provincial Genomics Data Center, BGl Research, Beishan Road, Yantian District, Shenzhen 518083, China; BGI Research, Shenzhen 518083, China; Guangdong Provincial Genomics Data Center, BGl Research, Beishan Road, Yantian District, Shenzhen 518083, China; GigaScience Press, BGI Research, Shenzhen 518083, China; GigaScience Press, BGI Research, Shenzhen 518083, China; BGI Research, Shenzhen 518083, China; Guangdong Provincial Genomics Data Center, BGl Research, Beishan Road, Yantian District, Shenzhen 518083, China; GigaScience Press, BGI Research, Shenzhen 518083, China

**Keywords:** GigaDB, repository, updated system, datasets

## Abstract

GigaDB is a repository that links research articles with the underlying datasets, software, and metadata, helping to support open and reproducible research. As of February 2026, it contains 2,710 published datasets, covering 90.56 TB of data. Over the past year, the platform has been rebuilt to better support the growing scale of the repository and to improve data submission, management, discoverability, and reuse. The updated system is organized around four core modules: role-based permission control, dataset management, workflow management, and dataset retrieval. These improvements make submission and curation more efficient and transparent, strengthen access to published data and related resources, and provide a strong foundation for the future development of GigaDB services.

## Introduction

Modern scientific data are growing at an unprecedented scale and have become central to discovery, especially in artificial intelligence [1]. This makes robust academic data archiving imperative for scientific progress and reproducibility. However, academic data in the life sciences currently remain highly fragmented, inconsistent in quality, and challenging to reuse. Despite improvements in data sharing and data curation over the past decade, significant disparities remain across disciplines in data availability and quality [2].

To address these challenges, the global scientific community has developed a range of initiatives and platforms to improve the visibility, stewardship, and reuse of research data. Repository registries such as FAIRsharing, OpenDOAR, and re3data enhance the discoverability of scholarly data resources across disciplines [3–5]. In parallel, many domain databases implement the FAIR data principles—Findable, Accessible, Interoperable, and Reusable—including Sequence Read Archive (SRA), CNSA, and Genome Sequence Archive (GSA) [6–8]. However, these resources primarily function as primary archives for raw sequencing data, which are often extremely large and difficult to interpret without sufficient contextual information.

At the same time, the traditional publishing model remains largely article-centric, and data deposition is frequently decoupled from publication. As a result, substantial amounts of research data are dispersed across personal devices or heterogeneous, non-standard platforms, with weak links to core research outputs such as articles, software tools, and analysis workflows. This fragmentation hinders verification and reproducibility, reduces the efficiency of data reuse, and contributes to duplicated effort, ultimately limiting the translation of research data into broader scientific and societal impact. Therefore, dedicated repositories that archive not only raw data but also processed data and research context are essential for improving usability and enabling downstream applications.

Within this context, GigaDB [9] has emerged as an important resource for archiving and retrieving large-scale datasets, particularly those underpinning scientific publications [9]. Launched prior to the GigaScience journal in 2011, GigaDB was designed to address the limitations of making processed data publicly and permanently accessible, as well as ensuring the availability of essential codes for data processing and table/figure generation to maximize the reproducibility of research [10]. It provides a portal for supporting data and tools when suitable community repositories are unavailable, thereby reinforcing open-data principles. Since its inception, GigaDB has demonstrated consistent growth in data volume, discoverability, and reusability, evolving to meet the expanding needs of multidisciplinary scientific research.

To further enhance data openness and management services, recent enhancements to GigaDB have centered on upgrading the data submission and curation systems. Here, a substantial increase in the volume and diversity of datasets is reported, as well as the latest features and services implemented in GigaDB. These updates aim to better meet global demand for open sharing of scientific research data, improve the efficiency and quality of the platform service and management ability, and also facilitate future iterations of the database.

## Overview of GigaDB

The GigaDB data publishing platform is a multi-user, collaborative open-data management system with role-based access control (RBAC). It employs a decoupled front-end/back-end architecture and fine-grained permission management to enable end-to-end digital workflow management, including dataset submission, format validation, data curation and editing, DOI assignment, CC0 waiver management, and audit logging. Together, these components support the platform’s core model of integrated publishing.

**Figure 1:**
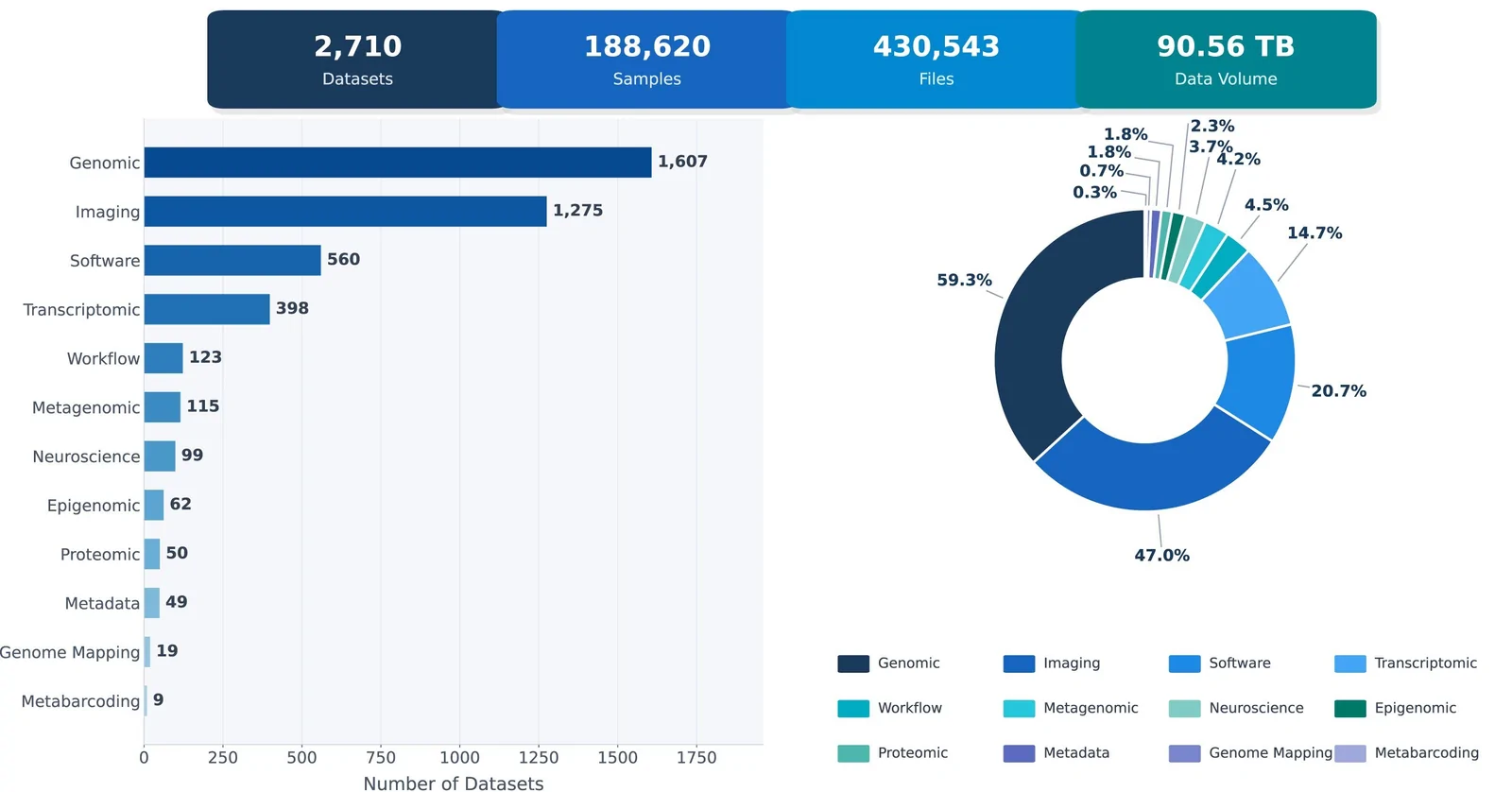
Data statistics of GigaDB. The upper panel shows the number of the datasets, samples, files and data volume in GigaDB. In the lower part, the left panel shows the number of datasets in different data types; the right panel shows the percentage of each data type.

GigaDB has demonstrated significant growth in both the volume and diversity of datasets it hosts. As of February 2026, GigaDB has archived 2,710 datasets comprising a total of 90.56 terabytes of data, encompassing 188,620 biological samples and 430,543 individual files (Fig. 1). The repository hosts a diverse array of heterogeneous data types, including genomic, imaging, metabolomic and lipidomic, epigenomic, metagenomic, and software datasets. Overall, GigaDB exemplifies a modern open-data repository that supports large-scale biological data dissemination and fosters collaborative scientific discovery.

### New system of GigaDB

To address the growing demands of modern data publishing, the GigaDB system has been fundamentally rebuilt around four interconnected core modules: role permission control, dataset management, operation flow, and dataset retrieval. The primary aim of this restructuring is to establish a secure, transparent, and highly efficient end-to-end pipeline for data curation and publication.

**Role permission control:** The foundation of the system’s data security relies on a robust RBAC model. By enforcing fine-grained permission management and strict operational isolation between user types (e.g., researchers and data curators), the system actively prevents unauthorized access and accidental modifications. Researchers are strictly isolated to manage only their own pre-publication datasets, while curators are equipped with the global administrative privileges necessary for comprehensive system maintenance and data governance. This architecture serves as a crucial first line of defense, ensuring the uncompromised integrity and confidentiality of research data prior to publication.

**Dataset management:** To ensure maximum data flow transparency, a state-machine logic model has been implemented that tracks datasets across four distinct lifecycle stages: submitted, processing, approved, and published. This structured approach standardizes the collaborative interface between submitting researchers and internal curators, effectively eliminating information silos and communication errors. Crucially, it guarantees that no dataset can bypass the rigorous editorial review process before public release, thereby upholding the highest standards of data quality, safety, and scientific reliability.

**Operation flow:** A lightweight workflow engine has been introduced to automate and standardize the trajectory from initial submission to final publication. This module transforms historically decentralized, manual workflows into a highly visible, traceable, and automated pipeline. To enforce data provenance and security at the source, the system employs a dual-authentication mechanism requiring both standard account credentials and a unique manuscript ID. This strictly verifies user permissions, restricting the initiation of dataset submissions exclusively to authenticated researchers and preventing unauthorized data entry. Downstream, the module actively mitigates human error by augmenting the assigned curator’s manual review with an automated secondary system validation. During this review stage, the system automatically verifies the dataset’s compatibility with the CC0 public domain waiver and its adherence to platform submission standards before allowing progression. Coupled with automated email triggers at critical transition nodes, this hybrid approach of human oversight and system-enforced compliance significantly accelerates cross-role collaboration while ensuring verifiable data governance.

**Dataset retrieval:** Designed to serve the global scientific community, this module provides robust, multi-dimensional search capabilities to maximize data discoverability. GigaDB has established a multi-dimensional standard system for metadata management, primarily manifested in two aspects: adherence to major standards and principles and database architecture design. Regarding international general standards and principles, it ensures the professionalism and discoverability of data by adhering to GSC standards, establishes a standardized citation mechanism through DataCite DOI and DCC best practices, and follows the FAIR principles while adopting CC0 waivers to maximize the promotion of open data sharing. In terms of database architecture design, the core of its database architecture is a fully scalable database schema, which draws references from other well-established bioinformatics systems, such as the metadata model of NCBI’s SRA and the format of the ISA infrastructure, thereby ensuring the professionalism and interoperability of the system design. Users can efficiently locate datasets using key metadata parameters (e.g., titles, DOIs, authors, and research fields), directly supporting the FAIR data principles. A built-in compliance screening mechanism guarantees that all surfaced data adhere to legal sharing frameworks, mitigating copyright risks. Furthermore, comprehensive logging of search and access activities ensures full traceability, successfully balancing the mandate for open scientific sharing with stringent data security governance.

### Dataset submission and curation

GigaDB has moved from a traditional workflow, in which authors and the data curation team communicated offline to prepare dataset records, to an online submission and curation system. In the earlier model, much of the metadata collection and dataset preparation relied on direct exchanges between curators and authors. The new approach allows authors to submit their dataset information through an online process, making data submission more structured, consistent, and easier to manage.

**Figure 2:**
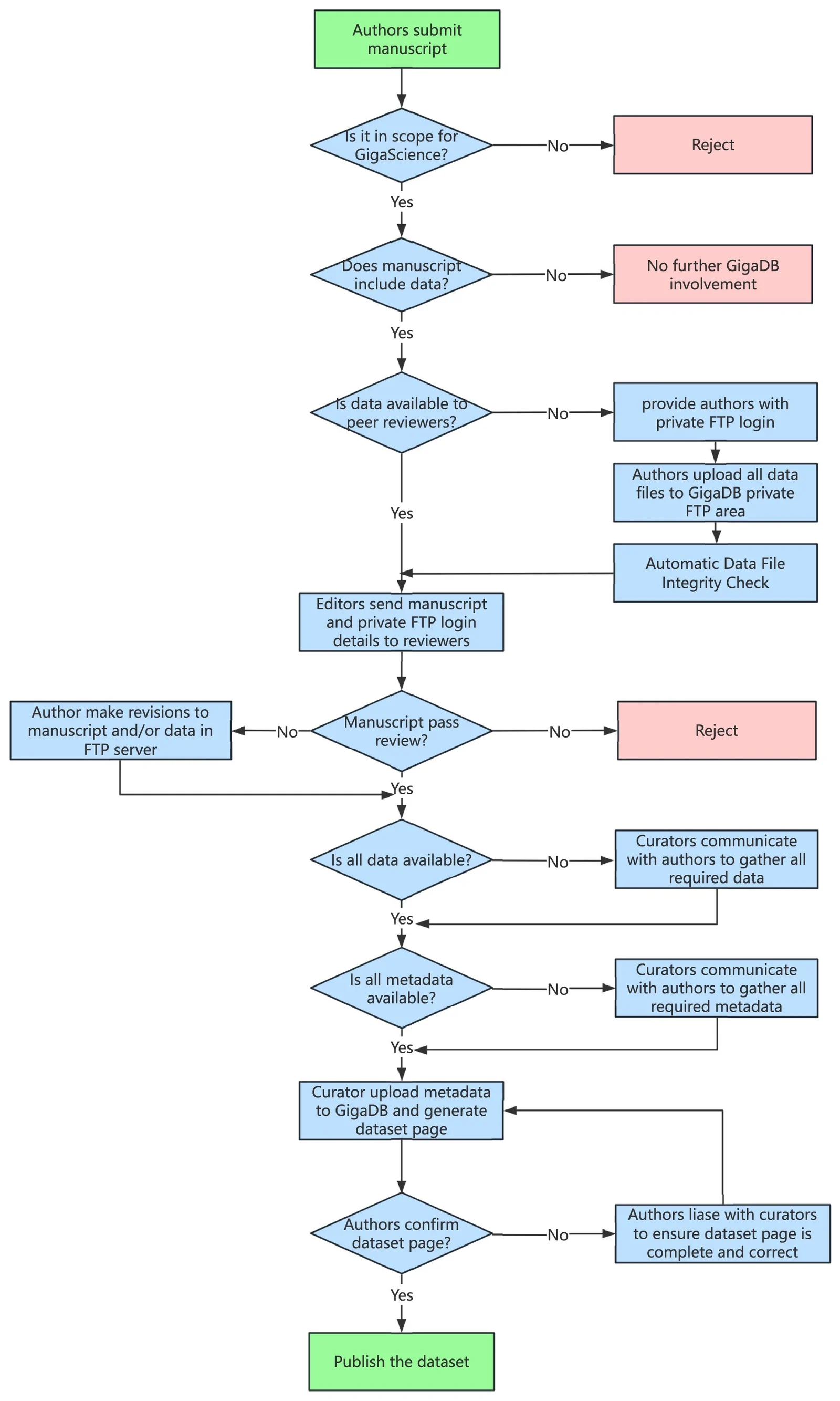
Pipeline of data submission and curation in GigaDB. Adapted from the GigaDB guidelines. https://gigadb.org/guide [11].

Once a manuscript is submitted to GigaScience, the journal editor will evaluate the manuscript from the perspective of whether it falls within the journal’s scope and whether the supporting data are sufficient for review. For submissions that include datasets, GigaDB facilitates confidential data access during peer review by providing authors with private FTP credentials when needed, allowing files to be deposited in a secure pre-publication space and shared with reviewers confidentially by the editorial team. Authors may update these files during the review process in response to editorial or reviewers’ comments. For manuscripts that will be ultimately accepted, curators then need to verify the correctness and completeness of both the submitted data files and the accompanying metadata, requesting any missing materials or clarifications from authors as necessary. Once all required information has been obtained, the metadata file will be uploaded to GigaDB, and a dataset preview page will be generated for authors. Following any final corrections and author confirmation, the dataset will be published in GigaDB with a permanent DOI and linked to the corresponding article (Fig. 2).

Compared to the previous version, this update provides seamless integration and automation for information and workflows across the entire dataset lifecycle. After submitting to GigaScience, authors will not need to retype the title, authors, and other core information in GigaDB. By simply entering their manuscript ID, the system will automatically retrieve this information from the journal’s database. During dataset transfer, authors upload files to temporary storage using private FTP credentials. The backend system will automatically validate file integrity and formats. Once approved by the curator, the dataset will be automatically moved to a protected archival directory and published. This process requires no manual intervention from curators and IT staff, eliminating delays and potential human errors. All steps, from metadata syncing to review notifications, will be recorded in permanent logs with timestamps and user roles. This end-to-end audit trail ensures full transparency for the entire research process.

This online workflow makes the submission and curation process more efficient and transparent. It reduces the need for repeated offline communication, helps standardize metadata collection, and ensures that datasets are ready for release alongside the corresponding article. As a result, the system improves both the author experience and curator efficiency.

## Discussion

As a critical infrastructure integrated into the GigaScience publishing workflow, GigaDB code is implemented on the dedicated GitLab platform operated by GigaScience Press, which seamlessly integrates code repositories, continuous integration/continuous deployment pipelines, code review mechanisms, issue tracking systems, container registries, and security scanning functionalities. This platform significantly enhances the security, standardization, and automation levels of the research and development workflow. The code is managed centrally within a dedicated environment that preserves comprehensive historical records, thereby facilitating auditing processes and enabling rollback capabilities, as well as supporting standardized and reusable deployment procedures. Furthermore, the system autonomously conducts testing, code scanning, and security assessments, achieving end-to-end automation. This approach mitigates the risk of data leakage, enhances collaborative efficiency, and minimizes instability caused by network fluctuations. GigaDB’s core value lies in dataset archiving, editorial review, and DOI assignment; the significance of this reconstruction lies not only in improving system performance but also in strengthening the relationship between the data platform and journal publishing. For the author, this means a clearer submission path, lower operational burden, and a shorter feedback chain. For editors and reviewers, this transformation facilitates the standardization of metadata and the automation of routine tasks, significantly increasing review efficiency while reducing the costs of manual coordination.

Building upon the transition to online submissions, automated workflows, and traceable audit logs, GigaDB’s future development will remain firmly aligned with its mission to support academic publishing. First, the platform will continue to refine data archiving protocols across diverse dataset types. These efforts are essential to improving data comprehensibility, interoperability, and long-term machine-readability. Second, the platform will progressively enhance its automated quality control pipeline. This will better support the peer review process. Third, through deeper technical integration and strategic collaboration with global research institutions and complementary databases, GigaDB aims to expand both the scope and richness of its data ecosystem for open scientific data sharing. Collectively, these initiatives will further reinforce GigaDB’s commitment to advancing a reproducible, verifiable, and reusable open science framework.

## Supplementary Material

giag047_Authors_Response_To_Reviewer_Comments_original_submission

giag047_GIGA-D-26-00110_original_submission

giag047_GIGA-D-26-00110_revision_1

giag047_Reviewer_1_Report_original_submission Reviewer 1 -- 4/9/2026
