# Correction to: ‘GigaDB: a redesigned repository for data publishing and management’

**DOI:** 10.1093/gigascience/giag085

**Published:** 2026-08-20

This is a correction to: Xiaoqiang Li, Cong Hua, Qian Yue, Zhiyong Li, Jiawei Tong, Ziheng Luo, Tao Yang, Lijin You, Hongfang Zhang, Dongni Ma, Xiaofeng Wei, Hongling Zhou, GigaDB: a redesigned repository for data publishing and management, *GigaScience*, Volume 15, 2026, giag047, https://doi.org/10.1093/gigascience/giag047.

In the original version of the article, the formal launch year of GigaDB was incorrectly stated as 2009, not 2011. Additionally, the Figure 2 caption should have included that the figure was adapted from the GigaDB guidelines [1]. The paper has been updated accordingly, and the authors apologize for the errors.
