# Supplementary material for: GigaDB: a redesigned repository for data publishing and management
Source: Gigascience. 2026 Apr 16;15:giag047. doi: 10.1093/gigascience/giag047 (PMC13201076; doi:10.1093/gigascience/giag047)
Supplement: giag047_GIGA-D-26-00110_original_submission [file giag047_giga-d-26-00110_original_submission.pdf]

GigaScience

GigaDB: a redesigned repository for data publishing and management

--Manuscript Draft--

**Manuscript Number:**	GIGA-D-26-00110
**Full Title:**	GigaDB: a redesigned repository for data publishing and management
**Article Type:**	Commentary
**Funding Information:**	High-quality Science and Technology Journal Construction Project of Guangdong Province (No. 2025B1212100003) — Not applicable<br>High-quality Science and Technology Journal Construction Project of Guangdong Province (No. 2025B1212070003) — Not applicable
**Abstract:**	GigaDB is a repository that links research articles with the underlying datasets, software, and metadata, helping to support open and reproducible research. As of February 2026, it contains 2,710 published datasets, covering 90.56 TB of data. Over the past year, we have rebuilt the platform to better support the growing scale of the repository and to improve data submission, management, discoverability, and reuse. The updated system is organized around four core modules: role-based permission control, dataset management, workflow management, and dataset retrieval. These improvements make submission and curation more efficient and transparent, strengthen access to published data and related resources, and provide a strong foundation for future development of GigaDB services.
**Corresponding Author:**	Hongling Zhou<br><br>CHINA
**Corresponding Author Secondary Information:**	
**Corresponding Author's Institution:**	
**Corresponding Author's Secondary Institution:**	
**First Author:**	Xiaoqiang Li
**First Author Secondary Information:**	
**Order of Authors:**	Xiaoqiang Li
	Cong Hua
	Zhiyong Li
	Jiawei Tong
	Ziheng Luo
	Tao Yang
	Lijin You
	Hongfang Zhang
	Dongni Ma, Ph.D.
	Xiaofeng Wei
	Hongling Zhou
**Order of Authors Secondary Information:**	
**Additional Information:**	

Powered by Editorial Manager® and ProduXion Manager® from Aries Systems Corporation

Question	Response
Are you submitting this manuscript to a special series or article collection?	No
**Experimental design and statistics**<br><br>Full details of the experimental design and statistical methods used should be given in the Methods section, as detailed in our Minimum Standards Reporting Checklist. Information essential to interpreting the data presented should be made available in the figure legends.<br><br>Have you included all the information requested in your manuscript?	Yes
**Resources**<br><br>A description of all resources used, including antibodies, cell lines, animals and software tools, with enough information to allow them to be uniquely identified, should be included in the Methods section. Authors are strongly encouraged to cite Research Resource Identifiers (RRIDs) for antibodies, model organisms and tools, where possible.<br><br>Have you included the information requested as detailed in our Minimum Standards Reporting Checklist?	Yes
**Availability of data and materials**<br><br>All datasets and code on which the conclusions of the paper rely must be either included in your submission or deposited in publicly available repositories (where available and ethically appropriate), referencing such data using a unique identifier in the references and in the "Availability of Data and Materials" section of your manuscript.	Yes

Have you have met the above requirement as detailed in our Minimum Standards Reporting Checklist?	
GigaScience has policies and guidelines in place for the use of generative AI-writing tools such as ChatGPT. If you have used such writing tools to assist with writing the manuscript this must be declared and cited in the text. Authors should not list AI-writing tools and other AI-assisted technologies as an author or co-author and should acknowledge that they are fully responsible for text generated or refined by AI-writing tools.<p><br><br>A summary of use (particularly in the introduction or among methods) needs to be included at the end of the paper, and the outputs should also be included as a supplementary file hosted in GigaDB or other open repositories. Please read our guidelines for more information. <p><br><br>By submitting to GigaScience, you are aware of the journal's AI-writing tools policy, and if you have declared use of such tools below, you have acknowledged this where appropriate in your manuscript and have made a summary of use and outputs available. <p><br><b>AI-assisted writing tools have been used in the preparation of this manuscript?	No

Powered by Editorial Manager® and ProduXion Manager® from Aries Systems Corporation

GigaDB: a redesigned repository for data publishing and management

Xiaoqiang Li[1], Cong Hua[1], Zhiyong Li[1], Jiawei Tong[1], Ziheng Luo[1], Tao Yang[2], Lijin You[2], Hongfang Zhang[3], Dongni Ma[3], Xiaofeng Wei[1,2,*], Hongling Zhou[3,*]

[1]BGI Research, Shenzhen 518083, China
[2]Guangdong Provincial Genomics Data Center, BGl Research, Beishan Road, Yantian District, Shenzhen 518083, China
[3]GigaScience Press, BGI Research, Shenzhen 518083, China

*Corresponding authors: Hongling Zhou, hongling@gigasciencejournal.com; Xiaofeng Wei, weixiaofeng@genomics.cn

Abstract

GigaDB is a repository that links research articles with the underlying datasets, software, and metadata, helping to support open and reproducible research. As of February 2026, it contains 2,710 published datasets, covering 90.56 TB of data. Over the past year, we have rebuilt the platform to better support the growing scale of the repository and to improve data submission, management, discoverability, and reuse. The updated system is organized around four core modules: role-based permission control, dataset management, workflow management, and dataset retrieval. These improvements make submission and curation more efficient and transparent, strengthen access to published data and related resources, and provide a strong foundation for future development of GigaDB services.

Introduction

Modern scientific data is growing at an unprecedented scale and has become central to discovery, especially in artificial intelligence (AI)[1]. This makes robust academic data archiving imperative for scientific progress and reproducibility. However, academic data in the life sciences currently remains highly fragmented, inconsistent in quality, and challenging to reuse. Despite

improvements in data sharing and data curation over the past decade, significant disparities remain across disciplines in data availability and quality[2].

To address these challenges, the global scientific community has developed a range of initiatives and platforms to improve the visibility, stewardship, and reuse of research data. Repository registries such as FAIRsharing, OpenDOAR, and re3data enhance the discoverability of scholarly data resources across disciplines[3–5]. In parallel, many domain databases implement the FAIR Data Principles—Findable, Accessible, Interoperable, and Reusable—including SRA, CNSA, and GSA[6–8]. However, these resources primarily function as primary archives for raw sequencing data, which are often extremely large and difficult to interpret without sufficient contextual information.

At the same time, the traditional publishing model remains largely article-centric, and data deposition is frequently decoupled from publication. As a result, substantial amounts of research data are dispersed across personal devices or heterogeneous, non-standard platforms, with weak links to core research outputs such as articles, software tools, and analysis workflows. This fragmentation hinders verification and reproducibility, reduces the efficiency of data reuse, and contributes to duplicated effort, ultimately limiting the translation of research data into broader scientific and societal impact. Therefore, dedicated repositories that archive not only raw data but also the processed data and research context are essential for improving usability and enabling downstream applications.

Within this context, GigaDB (https://gigadb.org/) has emerged as an important resource for archiving and retrieving large-scale datasets, particularly those underpinning scientific publications[9]. Launched prior to the GigaScience journal in 2009, GigaDB was designed to address the limitations of making processed data publicly and permanently accessible, as well as ensuring the availability of essential codes for data processing and tables/figures generation to maximize the reproducibility of research[10]. It provides a portal for supporting data and tools when suitable community repositories are unavailable, thereby reinforcing open-data principles. Since its inception, GigaDB has demonstrated consistent growth in data volume, discoverability, and reusability, evolving to meet the expanding needs of multidisciplinary scientific research.

In order to further enhance data openness and management services. Recent enhancements to GigaDB have centered on upgrading the data submission and curation systems. Here, we report a

substantial increase in the volume and diversity of datasets, as well as the latest features and services implemented in GigaDB. These updates aim to better meet global demand for open sharing of scientific research data, improve the efficiency and the quality of the platform service and management ability, and also make the future iterations of the database easier.

Main text

Overview of GigaDB

GigaDB data publishing platform is a multi-user, collaborative open-data management system with role-based access control. It employs a decoupled front-end/back-end architecture and fine-grained permission management to enable end-to-end digital workflow management, including dataset submission, format validation, data curation and editing, DOI assignment, CC0 waiver management, and audit logging. Together, these components support the platform’s core model of integrated publishing.

GigaDB has demonstrated significant growth in both the volume and diversity of datasets it hosts. As of February 2026, GigaDB has archived 2,710 datasets comprising a total of 90.56 terabytes (TB) of data, encompassing 188,620 biological samples and 430,543 individual files (Fig. 1). The repository hosts a diverse array of heterogeneous data types, including genomic, imaging, metabolomic and lipidomic, epigenomic, metagenomic, and software datasets. Overall, GigaDB exemplifies a modern open-data repository that supports large-scale biological data dissemination and fosters collaborative scientific discovery

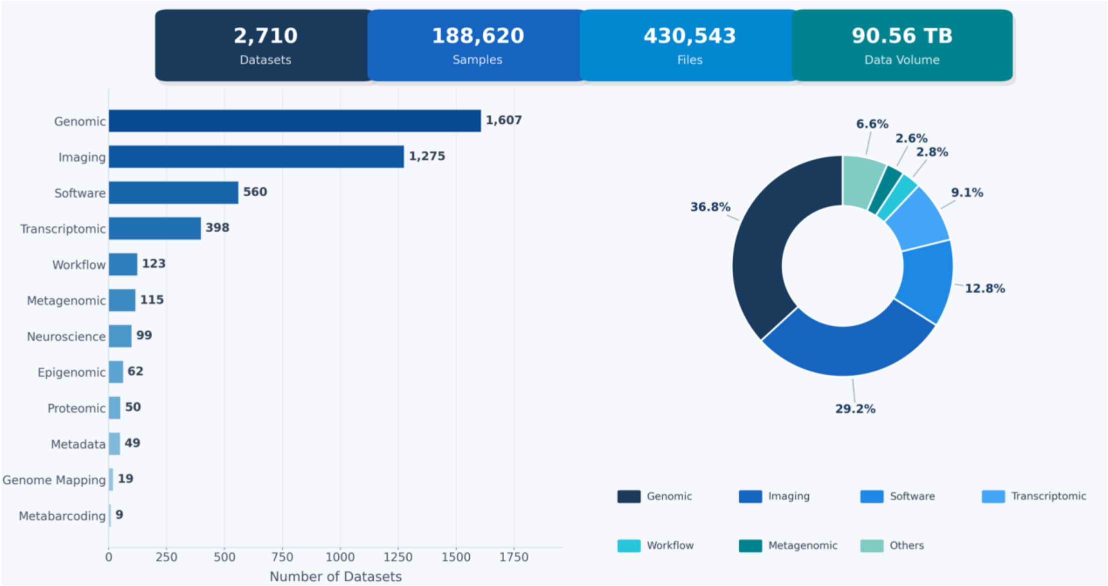

Fig 1. Data statistics of GigaDB.

New System of GigaDB

To address the growing demands of modern data publishing, we have fundamentally rebuilt the GigaDB system around four interconnected core modules: Role Permission Control, Dataset Management, Operation Flow, and Dataset Retrieval. Our primary aim in this restructuring is to establish a secure, transparent, and highly efficient end-to-end pipeline for data curation and publication.

Role Permission Control: The foundation of the system's data security relies on a robust Role-Based Access Control (RBAC) model. By enforcing fine-grained permission management and strict operational isolation between user types (e.g., researchers and data curators), the system actively prevents unauthorized access and accidental modifications. Researchers are strictly isolated to manage only their own pre-publication datasets, while curators are equipped with the global administrative privileges necessary for comprehensive system maintenance and data governance. This architecture serves as a crucial first line of defense, ensuring the uncompromised integrity and confidentiality of research data prior to publication.

Dataset Management: To ensure maximum data flow transparency, we implemented a state-machine logic model that tracks datasets across four distinct lifecycle stages: Submitted, Processing, Approved, and Published. This structured approach standardizes

the collaborative interface between submitting researchers and internal curators, effectively eliminating information silos and communication errors. Crucially, it guarantees that no dataset can bypass the rigorous editorial review process before public release, thereby upholding the highest standards of data quality, safety, and scientific reliability.

Operation Flow: We introduced a lightweight workflow engine to automate and standardize the trajectory from initial submission to final publication. This module transforms historically decentralized, manual workflows into a highly visible, traceable, and automated pipeline. To enforce data provenance and security at the source, the system employs a dual-authentication mechanism requiring both standard account credentials and a unique manuscript ID. This strictly verifies user permissions, restricting the initiation of dataset submissions exclusively to authenticated researchers and preventing unauthorized data entry. Downstream, the module actively mitigates human error by augmenting the assigned curator's manual review with an automated secondary system validation. During this review stage, the system automatically verifies the dataset's compatibility with the CC0 public domain waiver and its adherence to platform submission standards before allowing progression. Coupled with automated email triggers at critical transition nodes, this hybrid approach of human oversight and system-enforced compliance significantly accelerates cross-role collaboration while ensuring verifiable data governance.

Dataset Retrieval: Designed to serve the global scientific community, this module provides robust, multi-dimensional search capabilities to maximize data discoverability. Users can efficiently locate datasets using key metadata parameters (e.g., titles, DOIs, authors, and research fields), directly supporting the FAIR (Findable, Accessible, Interoperable, Reusable) data principles. A built-in compliance screening mechanism guarantees that all surfaced data adheres to legal sharing frameworks, mitigating copyright risks. Furthermore, comprehensive logging of search and access activities ensures full traceability, successfully balancing the mandate for open scientific sharing with stringent data security governance.

Dataset submission and curation

GigaDB has moved from a traditional workflow, in which authors and the data curation team

communicated offline to prepare dataset records, to an online submission and curation system. In the earlier model, much of the metadata collection and dataset preparation relied on direct exchanges between curators and authors. The new approach allows authors to submit their dataset information through an online process, making data submission more structured, consistent, and easier to manage.

Once a manuscript is submitted to *GigaScience*, the journal editor will evaluate the manuscript from the perspective of whether it falls within the journal's scope and whether the supporting data are sufficient for review. For submissions that include dataset, GigaDB facilitates confidential data access during peer review by providing authors with private FTP credentials when needed, allowing files to be deposited in a secure pre-publication space and shared with reviewers by the editorial team confidentially. Authors may update these files during the review process in response to editorial or reviewer' comments. For manuscripts that will be ultimately accepted, curators then need to verify the correctness and completeness of both the submitted data files and the accompanying metadata, request any missing materials or clarifications from authors as necessary. Once all required information has been obtained, the metadata file will be uploaded to GigaDB and a dataset pre-view page will be generated for authors. Following any final corrections and author's confirmation, the dataset will be published in GigaDB with a permanent doi and linked to the corresponding article (Fig. 2).

Compared to the previous version, this update provides seamless integration and automation for information and workflows across the entire dataset lifecycle. After submitting to GigaScience, authors will not need to re-type the title, authors, and other re-enter core information in GigaDB. By simply entering their Manuscript ID, the system will automatically pull this information from the journal's database. During dataset transfer, author upload files to temporary storage using private FTP credentials. The backend system will automatically validate file integrity and formats. Once approved by curator, the dataset will be automatically moved to a protected archival directory and published. This process requires no manual intervention from curators and IT staff, eliminating delays and potential human error. All steps, from metadata syncing to review notifications, will be recorded in permanent logs with timestamps and user roles. This end-to-end audit trail ensures full transparency for the entire research process.

This online workflow makes the submission and curation process more efficient and transparent.

It reduces the need for repeated offline communication, helps standardize metadata collection, and ensures that datasets are ready for release alongside the corresponding article. As a result, the system improves both the author experience and curator efficiency.

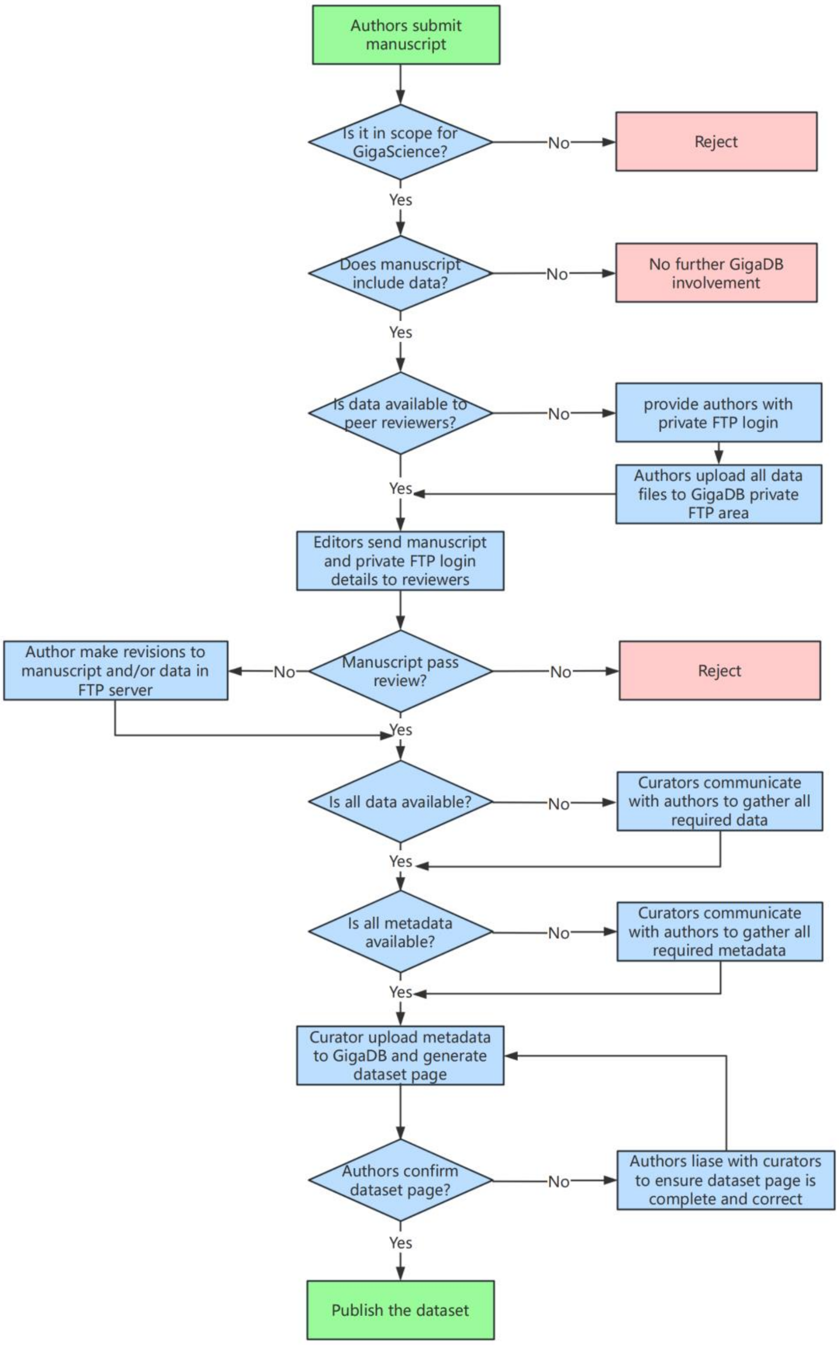

Figure 2 . Pipline of data submission and curation in GigaDB.

Discussion

As a critical infrastructure integrated into the GigaScience publishing workflow, GigaDB's core value lies in dataset archiving, editorial review, and DOI assignment. The significance of this reconstruction lies not only in improving system performance but also in strengthening the relationship between the data platform and journal publishing. For the author, this means a clearer submission path, lower operational burden, and a shorter feedback chain. For editors and reviewers, this transformation facilitates the standardization of metadata and the automation of routine tasks, significantly increasing review efficiency while reducing the costs of manual coordination.

Building upon the transition to online submissions, automated workflows, and traceable audit logs, GigaDB's future development will remain firmly aligned with its mission to support academic publishing. First, the platform will continue to refine data archiving protocols across diverse dataset types. These efforts are essential to improving data comprehensibility, interoperability, and long-term machine-readability. Second, the platform will progressively enhance its automated quality control pipeline. This will better support the peer review process. Third, through deeper technical integration and strategic collaboration with global research institutions and complementary databases, GigaDB aims to expand both the scope and richness of its data ecosystem for open scientific data sharing. Collectively, these initiatives will further reinforce GigaDB's commitment to advancing a reproducible, verifiable, and reusable open science framework.

Funding

This work was supported by the High-quality Science and Technology Journal Construction Project of Guangdong Province [No. 2025B1212100003] and [No. 2025B1212070003].

References

1. Wang H, Fu T, Du Y, Gao W, Huang K, Liu Z, et al.. Scientific discovery in the age of artificial

intelligence. *Nature*. 2023; doi: 10.1038/s41586-023-06221-2.

2. Tedersoo L, Küngas R, Oras E, Köster K, Eenmaa H, Leijen Ä, et al.. Data sharing practices and data availability upon request differ across scientific disciplines. *Scientific Data*. 2021; doi: 10.1038/s41597-021-00981-0.

3. Sansone S-A, McQuilton P, Rocca-Serra P, González-Beltrán A, Izzo M, Lister A, et al.. FAIRsharing as a community approach to standards, repositories and policies. *Nature Biotechnology*. 2019; doi: 10.1038/s41587-019-0080-8.

4. Pampel H, Vierkant P, Scholze F, Bertelmann R, Kindling M, Klump J, et al.. Making Research Data Repositories Visible: The re3data.org Registry. *PLOS ONE*. Public Library of Science; 2013; doi: 10.1371/journal.pone.0078080.

5. Moulaison HL, Dykas F, Gallant K. OpenDOAR Repositories and Metadata Practices. *D Lib Mag*. 212015;

6. Wang W, Tan C, Li L, Li X, Zhang L, Li X, et al.. The China National GeneBank Sequence Archive (CNSA) 2024 update. *Horticulture Research*. 2025; doi: 10.1093/hr/uhaf036.

7. Clough E, Barrett T, Wilhite SE, Ledoux P, Evangelista C, Kim IF, et al.. NCBI GEO: archive for gene expression and epigenomics data sets: 23-year update. *Nucleic Acids Research*. 2024; doi: 10.1093/nar/gkad965.

8. Chen T, Chen X, Zhang S, Zhu J, Tang B, Wang A, et al.. The Genome Sequence Archive Family: Toward Explosive Data Growth and Diverse Data Types. *Genomics, Proteomics & Bioinformatics*. 2021; doi: 10.1016/j.gpb.2021.08.001.

9. Sneddon TP, Si Zhe X, Edmunds SC, Li P, Goodman L, Hunter CI. GigaDB: promoting data dissemination and reproducibility. *Database*. 2014; doi: 10.1093/database/bau018.

10. Armit C, Tuli MA, Hunter CI. A Decade of GigaScience: GigaDB and the Open Data Movement. *GigaScience*. 2022; doi: 10.1093/gigascience/giac053.

Click here to access/download;Figure;Fig 1. Data statistics of GigaDB.pdf

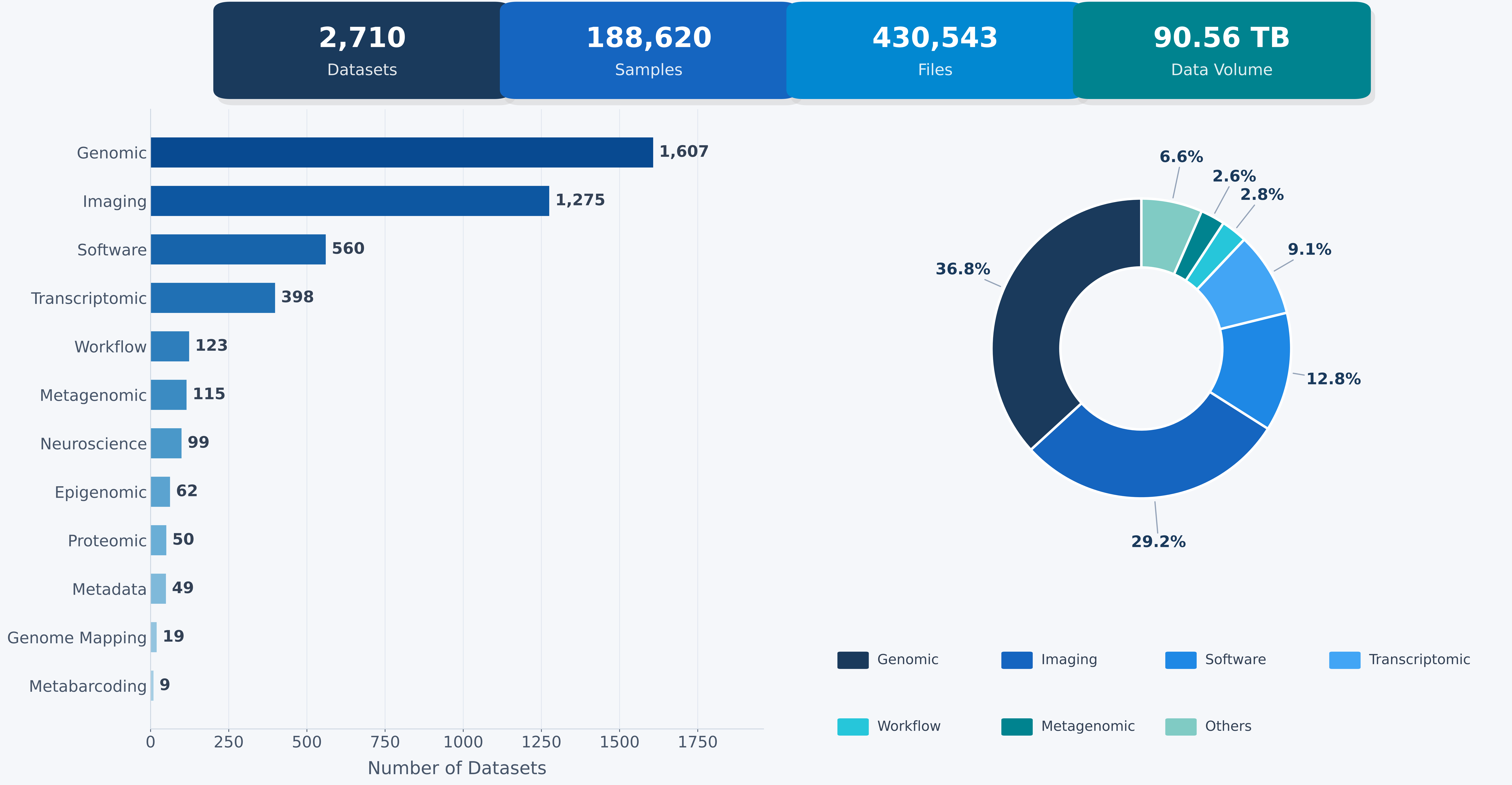

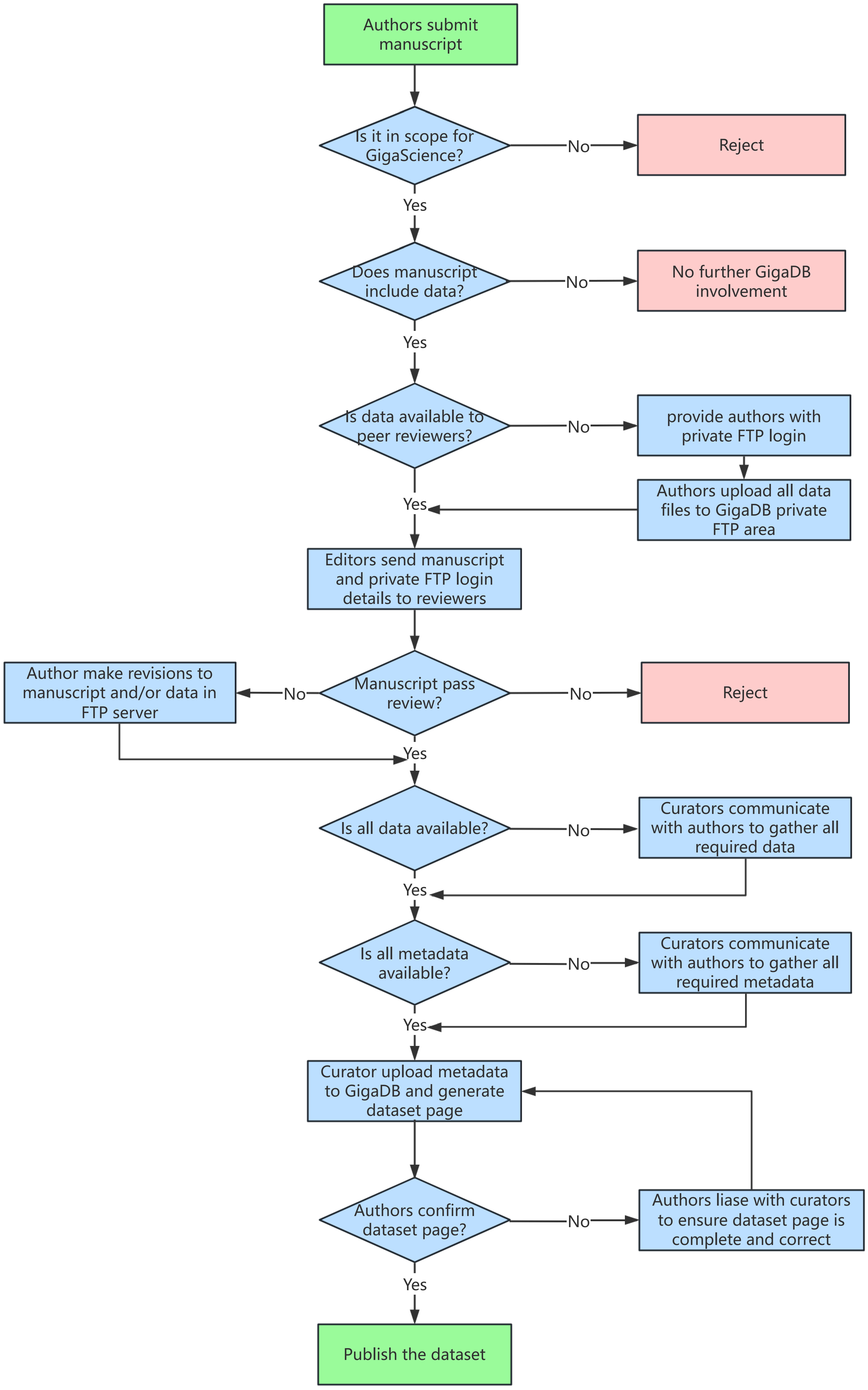
Authors submit manuscript
Is it in scope for GigaScience?
No
Reject
Yes
Does manuscript include data?
No
No further GigaDB involvement
Yes
Is data available to peer reviewers?
No
provide authors with private FTP login
Authors upload all data files to GigaDB private FTP area
Yes
Editors send manuscript and private FTP login details to reviewers
Manuscript pass review?
No
Author make revisions to manuscript and/or data in FTP server
No
Reject
Yes
Is all data available?
No
Curators communicate with authors to gather all required data
Yes
Is all metadata available?
No
Curators communicate with authors to gather all required metadata
Yes
Curator upload metadata to GigaDB and generate dataset page
Authors confirm dataset page?
No
Authors liase with curators to ensure dataset page is complete and correct
Yes
Publish the dataset
