# Supplementary material for: GigaDB: a redesigned repository for data publishing and management
Source: Gigascience. 2026 Apr 16;15:giag047. doi: 10.1093/gigascience/giag047 (PMC13201076; doi:10.1093/gigascience/giag047)
Supplement: giag047_Reviewer_1_Report_original_submission — Reviewer 1 -- 4/9/2026 [file giag047_reviewer_1_report_original_submission.pdf]

Reviewer Report

Title: GigaDB: a redesigned repository for data publishing and management

Version: Original Submission **Date:** 4/9/2026

Reviewer name: Reviewer 1

Reviewer Comments to Author:

This manuscript provides a solid overview of the GigaDB redesign, focusing on system architecture and the improved submission pipeline. As a data editor, I have a few minor suggestions to enhance the clarity, consistency, and alignment with FAIR principles before finalization.
Data Visualization (Figure 1)
Label Clarity: In the donut chart, the "Others" category accounts for 6.6%. It would be beneficial to list what is included in "Others" within the figure legend (e.g., Metadata, Genome Mapping, etc.) to match the bar chart on the left.
Technical Details & FAIR Compliance
Interoperability: You mention adherence to FAIR principles. It would strengthen the "Dataset Retrieval" section to explicitly mention the schema or ontologies used for metadata (e.g., Schema.org, Dublin Core, or specific biological ontologies).
Submission Pipeline (Figure 2)
Process Detail: In the flowchart, there is a step for "Authors upload all data files to GigaDB private FTP area." It might be helpful to clarify if the "automated secondary system validation" mentioned in the text happens immediately upon upload or after the curator's manual review.
Typo Check: In the text under Dataset submission and curation, there is a phrase: "...author upload files to temporary storage." This should be corrected to the plural: "authors upload files."
Discussion & Future Directions
Software Preservation: GigaDB is unique in hosting software/code. Briefly mention if the new system integrates with services like GitHub or Zenodo for long-term code archival.
Overall, the manuscript effectively communicates the technical leap from offline coordination to an integrated, transparent digital workflow. Implementing these minor adjustments will further improve the "discoverability and reuse" goals mentioned in your abstract.

Methods

Are the methods appropriate to the aims of the study, are they well described, and are necessary controls included? Choose an item.

Conclusions

Are the conclusions adequately supported by the data shown? Choose an item.

Reporting Standards

Does the manuscript adhere to the journal's guidelines on [minimum standards of reporting?]() Choose an item.

Choose an item.

Statistics

Are you able to assess all statistics in the manuscript, including the appropriateness of statistical tests used? Choose an item.

Quality of Written English

Please indicate the quality of language in the manuscript: Choose an item.

Declaration of Competing Interests

Please complete a declaration of competing interests, considering the following questions:

- Have you in the past five years received reimbursements, fees, funding, or salary from an organisation that may in any way gain or lose financially from the publication of this manuscript, either now or in the future?
- Do you hold any stocks or shares in an organisation that may in any way gain or lose financially from the publication of this manuscript, either now or in the future?
- Do you hold or are you currently applying for any patents relating to the content of the manuscript?
- Have you received reimbursements, fees, funding, or salary from an organization that holds or has applied for patents relating to the content of the manuscript?
- Do you have any other financial competing interests?
- Do you have any non-financial competing interests in relation to this paper?

If you can answer no to all of the above, write 'I declare that I have no competing interests' below. If your reply is yes to any, please give details below.

I declare that I have no competing interests.

I agree to the open peer review policy of the journal. I understand that my name will be included on my report to the authors and, if the manuscript is accepted for publication, my named report including any attachments I upload will be posted on the website along with the authors' responses. I agree for my report to be made available under an Open Access Creative Commons CC-BY license (http://creativecommons.org/licenses/by/4.0/). I understand that any comments which I do not wish to be included in my named report can be included as confidential comments to the editors, which will not be published.

I agree to the open peer review policy of the journal

To further support our reviewers, we have joined with Publons, where you can gain additional credit to further highlight your hard work (see: https://publons.com/journal/530/gigascience). On publication of this paper, your review will be automatically added to Publons, you can then choose whether or not to claim your Publons credit. I understand this statement.

Yes Choose an item.
